# Simultaneous saccharification and cofermentation of lignocellulosic residues from commercial furfural production and corn kernels using different nutrient media

**DOI:** 10.1186/1754-6834-4-22

**Published:** 2011-07-31

**Authors:** Yong Tang, Danqing Zhao, Carrasco Cristhian, Jianxin Jiang

**Affiliations:** 1 Department of Chemistry and Chemical Engineering, Beijing Forestry University, Beijing, China; 2 Department of Chemical Engineering, Lund University, P.O. Box 124, 221 00, Lund, Sweden

## Abstract

**Background:**

As the supply of starch grain and sugar cane, currently the main feedstocks for bioethanol production, become limited, lignocelluloses will be sought as alternative materials for bioethanol production. Production of cellulosic ethanol is still cost-inefficient because of the low final ethanol concentration and the addition of nutrients. We report the use of simultaneous saccharification and cofermentation (SSCF) of lignocellulosic residues from commercial furfural production (furfural residue, FR) and corn kernels to compare different nutritional media. The final ethanol concentration, yield, number of live yeast cells, and yeast-cell death ratio were investigated to evaluate the effectiveness of integrating cellulosic and starch ethanol.

**Results:**

Both the ethanol yield and number of live yeast cells increased with increasing corn-kernel concentration, whereas the yeast-cell death ratio decreased in SSCF of FR and corn kernels. An ethanol concentration of 73.1 g/L at 120 h, which corresponded to a 101.1% ethanol yield based on FR cellulose and corn starch, was obtained in SSCF of 7.5% FR and 14.5% corn kernels with mineral-salt medium. SSCF could simultaneously convert cellulose into ethanol from both corn kernels and FR, and SSCF ethanol yield was similar between the organic and mineral-salt media.

**Conclusions:**

Starch ethanol promotes cellulosic ethanol by providing important nutrients for fermentative organisms, and in turn cellulosic ethanol promotes starch ethanol by providing cellulosic enzymes that convert the cellulosic polysaccharides in starch materials into additional ethanol. It is feasible to produce ethanol in SSCF of FR and corn kernels with mineral-salt medium. It would be cost-efficient to produce ethanol in SSCF of high concentrations of water-insoluble solids of lignocellulosic materials and corn kernels. Compared with prehydrolysis and fed-batch strategy using lignocellulosic materials, addition of starch hydrolysates to cellulosic ethanol production is a more suitable method to improve the final ethanol concentration.

## Background

Of the available biofuels that can partly replace the use of liquid petroleum to reduce greenhouse-gas pollution, ethanol is currently the most widely used [1]. Starch and sugars from existing food crops are the main feedstocks for bioethanol production, because they are easy to use and cost-efficient [2]; however, it is expected that the supply of starch materials will become limited in the future [3]. Meanwhile, there is a growing ethical concern about the diversion of edible crops for this purpose [4,5]. Lignocelluloses are the most promising renewable resource for bioethanol production [6]. However, the requirement for depolymerisation makes ethanol production from them cost-inefficient [7,8]. Another limitation of cellulosic ethanol production is the difficulty of using a high solids-loading operation in simultaneous saccharification and fermentation (SSF), which limits the final ethanol concentration [9].

Nevertheless, ethanol production from lignocellulosic materials, especially low-cost waste materials, is receiving increased attention [10]. The utilization of waste agricultural and industrial materials will simultaneously

allow disposal of waste products, reduce the landfill problem, and result in production of valuable products. Potential raw materials for bioethanol include wheat straw in Europe, corn stover in the USA and straw in China [11]. Many industrial waste products have distinctive advantages over agricultural straw and woods for ethanol production. Recycled paper sludge can be used for the production of ethanol or other chemicals without being pretreated for bioconversion, because the paper raw materials already undergo extensive processing during the paper-making process [12]. Another example is furfural residue (FR), an industrial waste in China. Commercial furfural-production facilities use corncobs as feedstock. The corncobs are heated under acidic conditions to hydrolyze arabinoxylans (hemicelluloses) into sugars, and then those sugars are converted into furfural. The cellulose and lignin in the cobs are relatively stable under these conditions, so the residues left over after the furfural production are enriched in cellulose and lignin. FR can then used to produce bioethanol with simple pretreatment, and there are low amounts of pentose sugars to be converted. The furfural industry generated about 23 million tonnes of FR annually between 2006 and 2009 in China, but only a small amount of residue was recycled as boiler fuel. Ethanol production from FR would not only reduce environmental pollution, but also efficiently use the corncob material [13]. However, FR contains some inhibitors, specifically furfural and 5-hydroxymethylfurfural (5-HMF). Detoxification is therefore necessary for ethanol production from FR, and rinsing with water has been proven to be an effective detoxifying method [14].

Much research has been devoted to reducing the cost of cellulosic ethanol by developing a low-cost pretreatment method, very efficient hydrolysis, and efficient fermentative microorganisms [3,15,16]. Economic improvements can also be achieved by optimising the process parameters and using process-integration techniques [6]. Kim *et al *developed a novel starch-derived ethanol process using chemical and thermal treatment for conversion of non-starch polysaccharides in hulled barley into fermentable sugars [17]. A recent study on SSF of mixtures of wheat straw and wheat meal showed that SSF of the mixtures could also enhance ethanol production [18]. We confirmed that the use of mixed substrates is a promising method, because it could increase the final ethanol concentration and replace starch materials with lignocelluloses. The most likely reason for a high final yield in SSF of wheat straw and meal mixtures is the dilution of inhibitors, because wheat meal hydrolysate was added to the lignocellulosic stream and, according to the literature, this can decrease the inhibitory effects [18]. However, starch materials also contain cellulose and hemicellulose which probably also contributed to the high yield obtained in this study. Linde *et al. *[19] showed that, by adding enzymes to hydrolyze cellulose and hemicellulose in protein-rich residues (dried distillers grains with solubles; DDGS), a byproduct of dry-mill starch to ethanol production, the ethanol yield increased by 5%. When the DDGS were pretreated before SSF, ethanol yield was increased by up to 14%.

Further reductions in cost would also expected from the reduction of chemicals used in the process. Addition of various nutrients is crucial for the efficient fermentation of cellulosic ethanol, and these contribute significantly to the cost of large-scale productions. Other studies on starch materials have shown that starch hydrolysates such as wheat hydrolysates are potential supplements for ethanol production from lignocellulosic hydrolysates [20].

It does not seem economically feasible to hydrolyze cellulose and hemicellulose in starch materials by adding cellulase and β-glucosidase in starch ethanol-production processes; however, it might be feasible to add starch hydrolysates into the fermentation step using lignocelluloses as substrates. In this fermentation system, cellulase and β-glucosidase could then hydrolyze cellulose from both lignocellulosic and starch materials. Meanwhile, starch hydrolysates might be a source of some nutrients for yeasts, thereby decreasing the consumption of chemicals. Few studies have focused on the feasibility of such integration.

In the present work, the simultaneous saccharification and cofermentation (SSCF) of FR and corn kernels, which have different nutrient compositions, were carried out. We investigated the final ethanol concentration, yield, number of live yeast cells, and yeast-cell death ratio. Compared with the fed-batch strategy and the use of prehydrolysis in lignocellulosic materials, the advantage of SSCF process was first tested in terms of the final ethanol concentration and yield. SSCF results from high water-insoluble solids (WIS) FR and corn kernels are summarized. Finally, the feasibility of ethanol production by SSCF of FR and corn kernels without additional organic nutrients was also evaluated.

## Results and Discussion

### Ethanol production and reducing sugar concentrations in simultaneous saccharification and (co)fermentation

**Figure 1:**
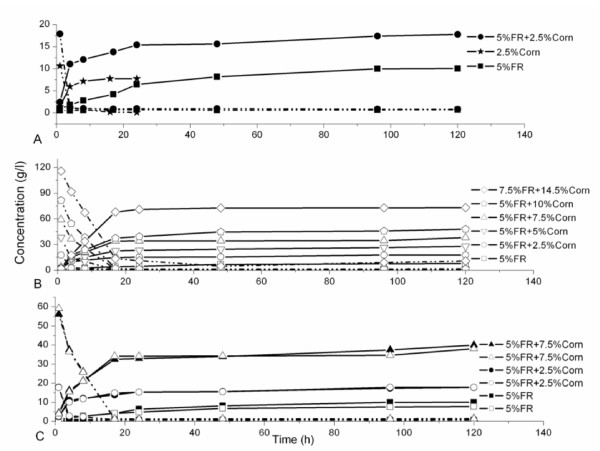
**Concentration of ethanol (solid line) and reducing sugars (dashed line) during simultaneous saccharification and (co)fermentation (SSF/SSCF) of furfural residue (FR) and corn kernels**. Closed symbols contained organic medium; open symbols contained mineral medium.

FR contains 42.2% cellulose, 38.7% lignin and 1.9% hemicellulose, and corn kernels contain 75.2% starch, 11.9% non-starch glucan and 17.7% washed solids residue (WSR). The concentration profiles of reducing sugars and ethanol during SSF/SSCF are shown in Figure 1. Similar concentration profiles were seen for both SSCF and SSF of corn kernels (Figure 1A). In the early stage, the overall kinetics were limited by the fermentation step because of the high concentrations of sugars, which were found to be parallel to the concentration of corn kernels at the beginning of SSCF. An increase in the concentration of corn kernels lengthened the time it took to deplete the sugars (Figure 1B, C). However, this was less than 48 h in all cases, which indicated that, for corn kernels, SSCF was as productive as SSF. The limiting step in the overall kinetics of SSF of FR was always enzymatic hydrolysis, and the reducing sugars stayed below 1.0 g/L.

SSF of lignocelluloses contains a lag phase because of the inhibition due to pretreatment byproducts and a change of medium. However, in our study, we did not see a lag phase in SSF of 5% FR, as the inhibitors had already been removed by the water-washing procedure. Ethanol production from SSCF of FR and corn kernels was faster than that from SSF of FR. The final ethanol concentration increased with increasing concentration of corn kernels in SSCF (Figure 1B), which is beneficial to reducing the cost of ethanol separation [21].

**Figure 2:**
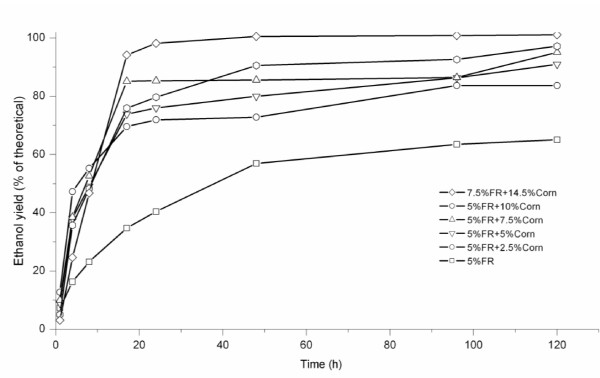
**The ethanol yield from simultaneous saccharification and (co)fermentation (SSF/SSCF) of furfural residue (FR) and corn kernels with mineral-salt medium**.

**Figure 3:**
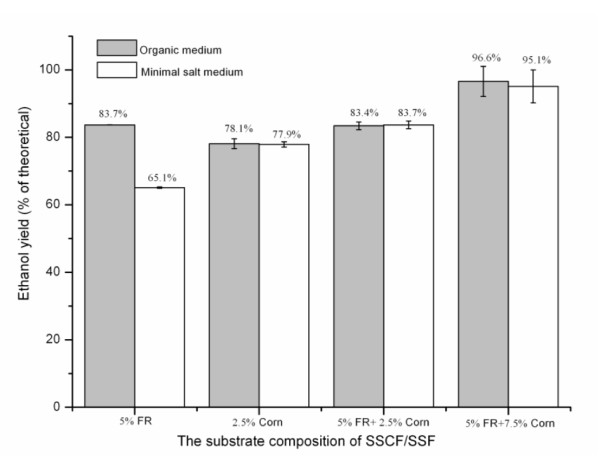
**The final ethanol yield from simultaneous saccharification and (co)fermentation (SSF/SSCF) of furfural residue (FR) and corn kernels with organic medium and mineral-salt medium**.

Comparing the ethanol-yield profiles of SSF/SSCF (Figure 2), the final yield from SSF of 5% FR with organic medium (Figure 3), was 83.7%, whereas that with mineral-salt medium was 65.1%, thus indicating that SSCF with organic medium was more appropriate to obtain higher yields. However, SSCF of 5% FR plus 2.5% corn kernels gave higher yields with mineral-salt medium than with organic medium, which was probably due to random errors in the analysis (Figure 3). The overall results indicated that organic nutrients were crucial for SSF of FR. Corn hydrolysate probably provides some of the nutrients required by the yeast, thus making the level of organic nutrients insignificant for SSCF and SSF of corn kernels. When SSF of 2.5% corn kernels was performed with Celluclast 1.5 L and Novozyme, the yield based upon corn starch would be ranged from 78% to 82%. However, it is not an economically feasible method in starch ethanol-production processes to add cellulase and β-glucosidase enzymes.

**Figure 4:**
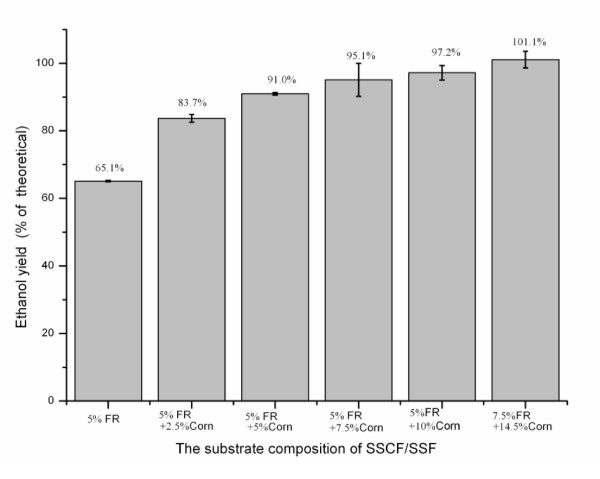
**The effect of corn concentration on the ethanol yield of simultaneous saccharification and cofermentation (SSCF) of furfural residue (FR) with mineral-salt medium at 120 hours**.

The final ethanol yield of SSCF increased with increasing concentration of corn kernels (Figure 4). SSCF of 5% FR plus 2.5% corn kernels produced higher ethanol yields than did SSF using either 5% FR or 2.5% corn kernels (without cellulosic enzymes) as substrate, indicating that the starch ethanol and cellulosic ethanol each promoted the production. The final yield (based on the theoretical yield of FR cellulose and cornstarch) was 101.1% for SSCF of 7.5% FR and 14.5% corn kernels with mineral-salt medium, because several nutrients and ethanol from WSR increased with increasing concentration of corn kernels in SSCF. The amount of non-starch glucan in corn kernels was 11.9%, and the yield (based upon the theoretical yield from FR cellulose, corn starch and corn non-starch glucan) was 90.3% for SSCF of 7.5% FR and 14.5% corn kernels.

By using a high concentration of substrates, a high final ethanol concentration was also obtained from cellulosic ethanol. However, a high concentration of lignocellulosic substrate (> 10%) would reduce the final ethanol yield because of higher viscosity and higher levels of inhibitor in the fermentation system [22]. Prehydrolysis can decrease the viscosity of a fermentation system with high WIS lignocelluloses, but SSF with prehydrolysis cannot reduce the presence of inhibitors and byproduct formation. Moreover, enzyme deactivation caused by prehydrolysis also lowers final yield [23]. Other studies on prehydrolysis have shown that the initial hydrolysis time is a significant factor affecting the yield of ethanol and other chemicals produced via biorefinery [24,25]. We found that compared with prehydrolysis, SSCF of lignocelluloses and corn hydrolysate improved the final ethanol concentration, while maintaining a high final ethanol yield. The reason for this might be that certain corn hydrolysates, with low viscosities and few inhibitors, could produce glucose at levels nearly 1.8 times as high as those from the same substrate concentration in the FR stream.

Fed-batch strategy is another potential method for performing SSF with high lignocellulose concentrations, because it can decrease the occurrence of stirring problems and the number of inhibitors in the fermentation system [26]. However, fed-batch SSF also requires high enzyme loading. The combination of enzyme and substrate feeding is necessary to maintain a high yield from fed-batch SSF or SSCF, and fed-batch SSF requires an optimal enzyme-feeding strategy, depending on the different substrate conditions [27,28]. According to Zhang MJ et al, fed-batch SSF reached a final dry-matter content of 25% w/v with a loading of 22.8 FPU cellulase/g glucan, 5 g/L dry yeast cells and a high ethanol concentration of 84.7 g/L was obtained after 96 hours, which corresponded to an overall ethanol yield of 79% [26]. An ethanol concentration and ethanol yield of 72.9 g/L and 100.8%, respectively, at 96 hours was obtained in this study.

Production of a greater amount of ethanol from corn cellulose is an advantage of the integration of cellulosic and starch ethanol, and is partly responsible for the high yield obtained from SSCF of cellulosic and starch materials. Lignin is the second most abundant component of FR, and is a key challenge to cost-effective depolymerisation when using lignocellulosic materials. Notably, WSR is difficult to market as animal fodder because of the lignin accumulation that occurs in the cellulosic and corn ethanol integration strategy [19]. High levels of delignified FR have been obtained to evaluate the enzymatic hydrolysis of cellulose residue [29]. Delignification of FR could provide a potential way to overcome this disadvantage in the future.

### The number of live yeast cells and the yeast-cell death ratio during simultaneous saccharification and (co)fermentation

**Figure 5:**
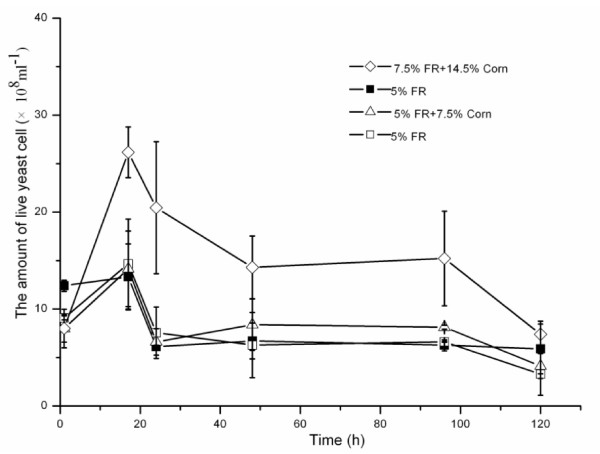
**The number of live yeast cells during simultaneous saccharification and (co)fermentation (SSF/SSCF) of furfural residue (FR) and corn kernels**. Closed symbols refer to organic medium, open symbols refer to mineral medium.

**Figure 6:**
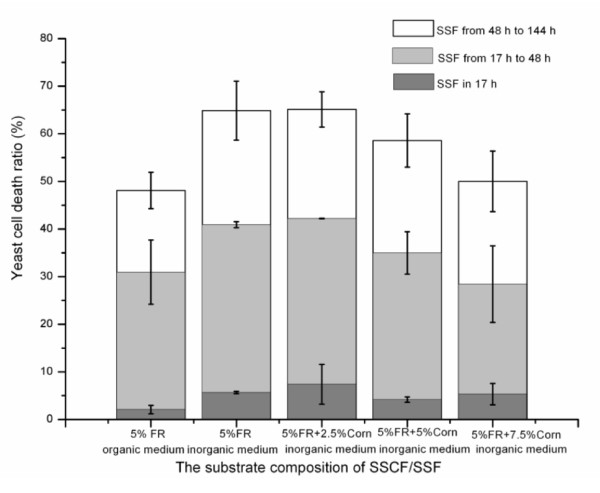
**The effect of corn concentration on yeast-cell death ratio during simultaneous saccharification and (co)fermentation (SSF/SSCF) of furfural residue (FR)**.

The number of live yeast cells increased in the first 17 h, and then decreased from 17 to 120 h (Figure 5). The yeast-cell death ratio decreased with increasing concentration of corn kernels during SSCF with mineral-salt medium (Figure 6), suggesting that there was more yeast proliferation with high concentration of corn kernels in SSCF. There were small differences in the number of live yeast cells between the organic and mineral-salt media during SSF of 5% FR. However, SSF of 5% FR with mineral-salt medium had a consistently higher yeast-cell death ratio than that with organic medium. The number of live yeast cells in SSCF of 7.5% FR and 14.5% corn kernels with mineral-salt medium was nearly twice that of SSF of 5% FR with organic medium, whereas similar yeast-cell death ratios were obtained for both groups. The number of live yeast cells increased in SSCF of FR plus corn kernels with mineral-salt medium, even at a concentration of 2.5% corn kernels. Moreover, the promoting effect that occurred between FR and corn kernels seemed to strengthen with increasing concentration of corn kernels.

Detoxification is always necessary for ethanol production from pretreated lignocelluloses to reduce the number of inhibitors [14]. Several studies have aimed to reduce the cost of detoxification by optimizing pretreatment parameters, cultivation procedures or other strategies [30-33]. Notably, inhibitors, specifically furfural and 5-HMF, exist in raw FR. Rinsing with water removed these inhibitors, but it is not an acceptable industrial solution to this problem. Another study on SSF of wheat straw and wheat meal mixtures sought to determine whether adding wheat-meal hydrolysate to the lignocellulosic stream would dilute the inhibitor concentration in the fermentation broth [18]; the study showed that addition of corn hydrolysate had a positive effect on yeast growth, which might be able to reduce freshwater consumption in washing procedures. Further studies on the effect of adding corn hydrolysate during detoxification of raw FR should be performed; addition of corn hydrolysate to SSF of FR could be an excellent strategy.

Lowering the yeast concentration can reduce the cost associated with yeast cultivation, because it is difficult to reuse yeast in SSF of lignocelluloses. However, a high yeast concentration may reduce lactic-acid production and enhance ethanol production. Stenberg *et al. *showed that SSF of softwood with 10 g/L yeast-cell mass had a higher overall ethanol yield (70%) than that with 2 g/L yeast-cell mass (64%) [34]. Other work has shown that using an inoculation rate of more than 3 × 10^7 ^yeast cells/ml reduced lactic-acid production significantly, while enhancing ethanol production [35]. Low yeast loading is also though to be inappropriate in fermentation systems with a high initial glucose concentration or hydrolysis rate, because this will lead to excess yeast reproduction early in fermentation. Interestingly, Narendranath and Power found no differences in the final ethanol yield with yeast inoculation rates of between 3 × 10^7 ^and 4 × 10^7 ^cells/ml [35]. In our study, SSCF of FR with 3.3 g/L yeast-cell mass corresponded to 8 × 10^7 ^cells/ml. It is interesting that a high initial yeast-cell mass at the start of SSF can reduce lactic-acid formation and avoid excessive yeast reproduction, but also reduces the ethanol yield. According to Linde *et al*., in SSF of 7.5% steam-pretreated barley straw with 20 FPU/g cellulose, the ethanol decreased from 22.4 g/L to 21.4 g/L after 120 h, with a decrease from 5 to 2 g/L of yeast; however, ethanol concentration increased from 18.5 to 19.0 g/L with enzyme loading of 10 FPU/g [23]. Therefore, it is crucial to choose the appropriate initial yeast-loading concentrations for the specific fermentation system. A study on continuous ethanol fermentation of cheese whey-powder solution showed that a feed-sugar concentration above 100 g/L had adverse effects on yeast growth and ethanol yield, and the optimal feed-sugar concentration ranged from 100 to 125 g/L [36]. We obtained an initial sugar concentration of 115.8 g/L from SSCF of 7.5% FR and 14.5% corn kernels, which, resulted in correspondingly high numbers of live yeast cells at 17 h.

The initial nitrogen concentration in the media strongly affects fermentation rate, but has no effect on the specific growth rates of *Saccharomyces cerevisiae *[37]. This could explain the small differences between organic and mineral-salt media in the number of live yeast cells obtained from SSF of 5% FR before 96 h. The high yeast-cell death ratios in SSF of 5% FR with mineral-salt medium indicate that sufficient organic nutrients can ensure yeast reproduction with low sugar consumption. High sugar concentrations (approximately 120 g/L) may promote yeast reproduction, according to the results of other studies [36,37], which results in rapid ethanol formation with loss of some glucose.

## Conclusions

SSCF of cellulosic and starch ethanol can produce ethanol at a high concentration and yield. The yield of SSCF with both FR and corn kernels was higher than that of SSF with either FR or corn kernels alone, and the ethanol yield of SSCF increased with increasing concentration of corn kernels. The production of additional ethanol from corn cellulose is beneficial for the integration of cellulosic and starch materials, which leads to a high ethanol yield in SSCF.

The integration of cellulosic ethanol with starch ethanol can also decrease chemical consumption. SSCF with mineral-salt medium produced the same ethanol yield as that with organic medium. The number of live yeast cells increased with increasing concentration of corn kernels in SSCF, while the yeast-cell death ratio decreased. Ethanol production from SSCF of a high substrate concentration of FR and corn kernels is therefore cost-efficient.

Addition of corn hydrolysate increased the final ethanol concentration while maintaining high ethanol yield compared with SSF of a high level of lignocellulosic substrate with prehydrolysis. Compared with the fed-batch strategy for SSF or SSCF of the high WIS lignocellulosic substrate, SSCF of lignocellulosic substrates and starch materials together efficiently produced ethanol without a need for high enzyme loading or organic nutrients. In the starch ethanol-production system, integration of cellulosic and starch ethanol reduced the consumption of starch materials compared with the addition of cellulase and β-glucosidase.

Lignin accumulation during the integration of cellulosic and starch ethanol makes it difficult to market the WSR as animal fodder, but studies on delignification could provide a potential way to overcome this disadvantage. More work is needed to evaluate the economic feasibility of the integration of FR and corn ethanol.

## Methods

### Raw material

Raw FR was kindly provided by Chunlei Company (Hebei Province, China). Raw FR, with an initial pH of 2 to 3, was dried at 60°C for 12 hours after being rinsed with water to neutral pH. Washing procedures partly remove inhibitors, including furfural and 5-HMF [14]. The corn kernels were kindly provided by Zhongliang Company (Beijing, China).

### Corn-starch hydrolysis

Corn-starch hydrolysis was performed in a 500-ml flask with 20% dry matter. Corn kernels were liquefied at 85°C for 2 h. Subsequent saccharification was performed at 60°C for 1 h (pH 4.0). The pH of the saccharification liquid was adjusted to 5.5 for SSF. Starch was liquefied and partially converted to glucose prior to performing SSF, to reduce the end-product inhibition of enzymes and the osmotic stress to yeast cells [18].

In one experiment, corn kernels were treated as above for the liquefaction step, but the saccharification step was allowed to go for 24 hours to completely convert all starch to glucose. The glucose was measured to determine the total starch in the kernels, and the remaining WSR was isolated, dried, weighed and analyzed to determine the non-starch polysaccharide levels in the corn kernels.

### Microorganism, inoculum and enzyme preparation

The microorganism used for fermentation was *S. cerevisiae *in the form of dry yeast (Angel Yeast Company Ltd, Yichang, China). Dry yeast was activated in 2% glucose solution at 36°C for 15 minutes, then at 34°C for 1 hour. Thermostable Alpha-amylase (150 U/g corn kernels) and glucoamylase (20 U/g) (Aoboxing Universeen Bio-Tech Company Ltd, Beijing, China) were used for corn liquefaction and saccharification, respectively. Cellulase (Celluclast 1.5 L; 75 filter paper units (FPU)/ml) and β-glucosidase (Novozyme 188; 43.9 IU/ml) enzyme preparations (both Novozymes A/S, Bagsvaerd, Denmark) were used for SSF/SSCF; the amount of Celluclast 1.5 L and Novozyme 188 added was 15 FPU and 17 IU per gram cellulose of FR respectively. SSF of corn kernels was performed without Celluclast 1.5 L and Novozyme 188 supplements.

### Simultaneous saccharification and (co)fermentation

The SSF/SSCF experiments were performed under nonsterile conditions in a 100-ml conical flask with a working weight of 60 g. In the anaerobic cultivations, each flask was equipped with a loop trap containing sterile glycerol. The concentrations ranged from 2.5% to 14.5% for corn kernels and from 5% to 7.5% for FR. Organic medium (yeast extract 1 g/L, (NH_4_)_2_HPO_4 _0.5 g/L, MgSO_4 _7H_2_O 0.5 g/L) and mineral-salt medium ((NH_4_)_2_HPO_4 _0.5 g/L, MgSO_4 _7H_2_O 0.5 g/L) were used. In each experiment, the conical flask was loaded with FR and fermentation medium, which were separately sterilized (121°C for 20 minutes). The enzymes, corn hydrolysate and yeast, with an initial cell mass concentration of 3.3 g dry matter per litre were then added to the conical flask directly. SSF/SSCF were performed at 38°C with an initial pH of 5.5. All experiments were agitated at 120 rpm in a shaking water-bath.

### Analytical methods

The content of cellulose and hemicellulose of raw materials were analyzed using procedures for the chemical analysis of wood and wood products [38]. The content of acid-insoluble lignin was determined according to the Tappi method (T 222 om-06) [39]. Samples were diluted by certain time points to determine the number of yeast cells by the blood-count method. 0.5 ml of sample diluent was mixed with isovolumetric 0.05% methylene blue, and subsequently the mixtures were left to stand for 3 minutes before the cell-death ratio was determined. The numbers of live yeast cells were calculated as follows:

The samples were filtered (0.22-μm pore) to detect the reducing sugars and ethanol. The total amount of reducing sugars was measured by the Somogyi-Nelson colorimetric method, with glucose as a standard [40]. Ethanol was determined using a gas chromatograph system (7890; Agilent Technologies, Beijing, China) equipped with a flame ionization detector and a stainless-steel column with length of 2.1 m and outer diameter of 2.6-mm. High-purity nitrogen was used as the carrier gas. The ethanol yield was calculated assuming that 1 g of cellulose or starch present in the liquid theoretically gave 0.568 g of ethanol,, and is expressed as the percentage of the theoretical yield based on FR cellulose and corn starch. Assays were performed in three repeated experiments, and mean values are presented.

## List of abbreviations

SSCF: simultaneous saccharification and cofermentation; SSF: simultaneous saccharification and fermentation; FR: furfural residue (rinsed with water); WIS: water-insoluble solid; DDGS: dried distillers grains with solubles (a protein-rich byproduct of dry-mill starch to ethanol production); WSR: the washed solids residue (after complete saccharification of corn kernels).

## Competing interests

The authors declare that they have no competing interests.

## Authors' contributions

YT and JJ designed and coordinated the study, YT and DZ carried out the experiments, and YT and CC analyzed the results. YT and DZ wrote the paper, and CC and JJ reviewed the paper. All authors read and approved the final manuscript.
